# Association of physical activity and fine motor performance in individuals with type 2 diabetes mellitus and/or non-alcoholic fatty liver disease

**DOI:** 10.1080/07853890.2023.2193422

**Published:** 2023-03-28

**Authors:** Ali A. Weinstein, Dung Ngo, Leyla de Avila, Jillian K. Price, Pegah Golabi, Patrick Austin, Carey Escheik, Lynn H. Gerber, Zobair M. Younossi

**Affiliations:** a Betty and Guy Beatty Center for Integrated Research, Inova Health System, Falls Church, VA, USA; b Department of Global and Community Health, George Mason University, Fairfax, VA, USA; c Department of Medicine, Center for Liver Disease, Inova Fairfax Hospital, Falls Church, VA, USA; d Inova Medicine, Inova Health System, Falls Church, VA, USA

**Keywords:** Mild cognitive impairment, obesity, steatosis, NAFLD

## Abstract

**Background:**

Fine motor performance may serve as an early warning sign for reduced cognitive function. Physical activity can help preserve cognitive function; however, the relationship between fine motor performance and physical activity is not well understood. Therefore, this study examined the relationship between fine motor performance and physical activity in individuals at risk for developing cognitive impairment (those with diabetes and/or non-alcoholic fatty liver disease (NAFLD)).

**Patients and methods:**

Individuals aged 25–69 with and without diabetes and NAFLD were enrolled. For this cross-sectional study, all participants completed the Human Activity Profile and fine motor performance tasks (Grooved Pegboard Test and Trail Making Test).

**Results:**

There were 93 participants in the study (NAFLD only (*n* = 29); diabetes + NAFLD (*n* = 34), controls (*n* = 30)). Individuals with both diabetes and NAFLD were less physically active and performed slower on the fine motor performance task. A statistically significant correlation was found between physical activity and motor speed among those with NAFLD only (*r* = 0.436, *p*<.05), which remained statistically significant after controlling for body mass index (*r* = 0.385; *p*<.05).

**Conclusions:**

This study suggests that those with diabetes + NAFLD have lower levels of physical activity and slower fine motor performance. The relationship between physical activity and fine motor performance was only statistically significant in the group of individuals with NAFLD only. Future research needs to explore the mechanisms that impact fine motor performance and physical activity in individuals at risk for mild cognitive impairment. Individuals with diabetes and/or NAFLD should be identified, advised and encouraged to engage in physical activity.

**Key Messages**
1. Those with NAFLD and T2DM have lower levels of physical activity and slower fine motor performance compared to controls and those with NAFLD only.
2. Future research needs to explore the mechanisms that impact fine motor performance and physical activity in those with T2DM with or without NAFLD.
3. Individuals with impaired fine motor performance should be identified and encouraged to engage in physical activity.

## Introduction

There has been a great deal of interest in understanding the relationships that exist among physical activity, cognitive performance and overall health and longevity [1–3]. As of now, no universally accepted measure is available for assessing mild (early) cognitive impairment (MCI), but some diagnoses have been reported to associate with a higher likelihood of MCI. Type 2 diabetes mellitus (T2DM) is one such condition [4, 5], specifically with performance decrements in the attention, concentration and processing speed domains [6–8]. A highly related diagnosis to T2DM is non-alcoholic fatty liver disease (NAFLD) [9], which is a growing public health problem and the most common chronic liver disease in the world [10,11]. However, only a small portion of research has investigated the specific relationship between NAFLD and cognitive performance [12,13]. T2DM and NAFLD have overlapping risk factors (i.e. obesity) and physical activity is related to reducing risks with both NAFLD [14] and T2DM [15].

An area of cognitive performance that may serve as an early warning sign for overall cognition is reduction in fine motor performance [16,17]. Fine motor performance refers to the coordinated, often purposeful activities of small muscles of the upper extremity and includes prehensile activities, handwriting and manually dexterous tasks. In addition, fine motor performance is related to engagement in physical activity [18–22]. Physical activity, which engages large muscle groups, is associated with arm and lower extremity activity rather than hand and wrist; it increases muscle strength, balance and mobility, thereby promoting function and longevity. In fact, high levels of physical activity can reduce the development of dementia [18, 19,22,23].

As mentioned above, physical activity is an important behaviour for both individuals with T2DM and those with NAFLD [24–26]. An exercise regimen, usually aerobic, is a treatment for these groups to improve metabolic and cardiovascular status. Adherence to this recommendation is notoriously difficult and may be especially ineffective in a population that prefers sedentary behaviours [27]. Determining if fine motor performance correlates with physical activity would help to further justify the prescription of exercise.

**Figure 1.**
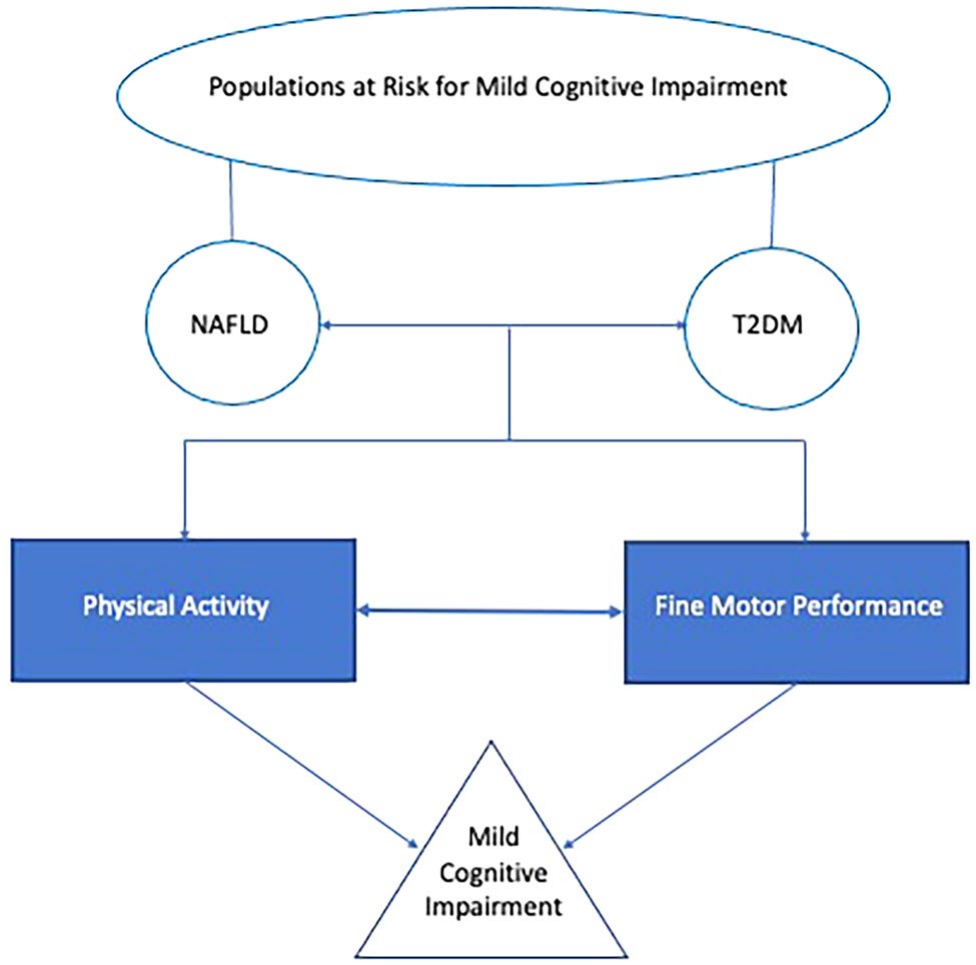
Conceptual model of study. Populations are represented with round shapes, behaviours/cognitive performance variables represented in rectangular shapes, and the condition (mild cognitive impairment) represented by a triangle. The variables that are the focus of the current investigation, physical activity and fine motor performance, are represented by filled shapes.

Therefore, the relationship between physical activity and fine motor performance is an important area to understand, specifically in individuals with NAFLD and/or T2DM, because these individuals are at a greater risk for developing MCI and dementia [28,29]. An area that is particularly challenging is the evaluation of early (mild) cognitive impairment due to a paucity of instruments designed to evaluate this. This is one reason we decided to pursue the use of fine motor performance, an objective, reliable, sensitive and possible early warning sign for cognitive decline [30], see Figure 1 for a conceptual model of the current investigation. The purpose of the current study was to assess the relationship between physical activity and fine motor performance in individuals at risk for developing MCI (individuals with T2DM and/or NAFLD).

## Methods

**Figure 2.**
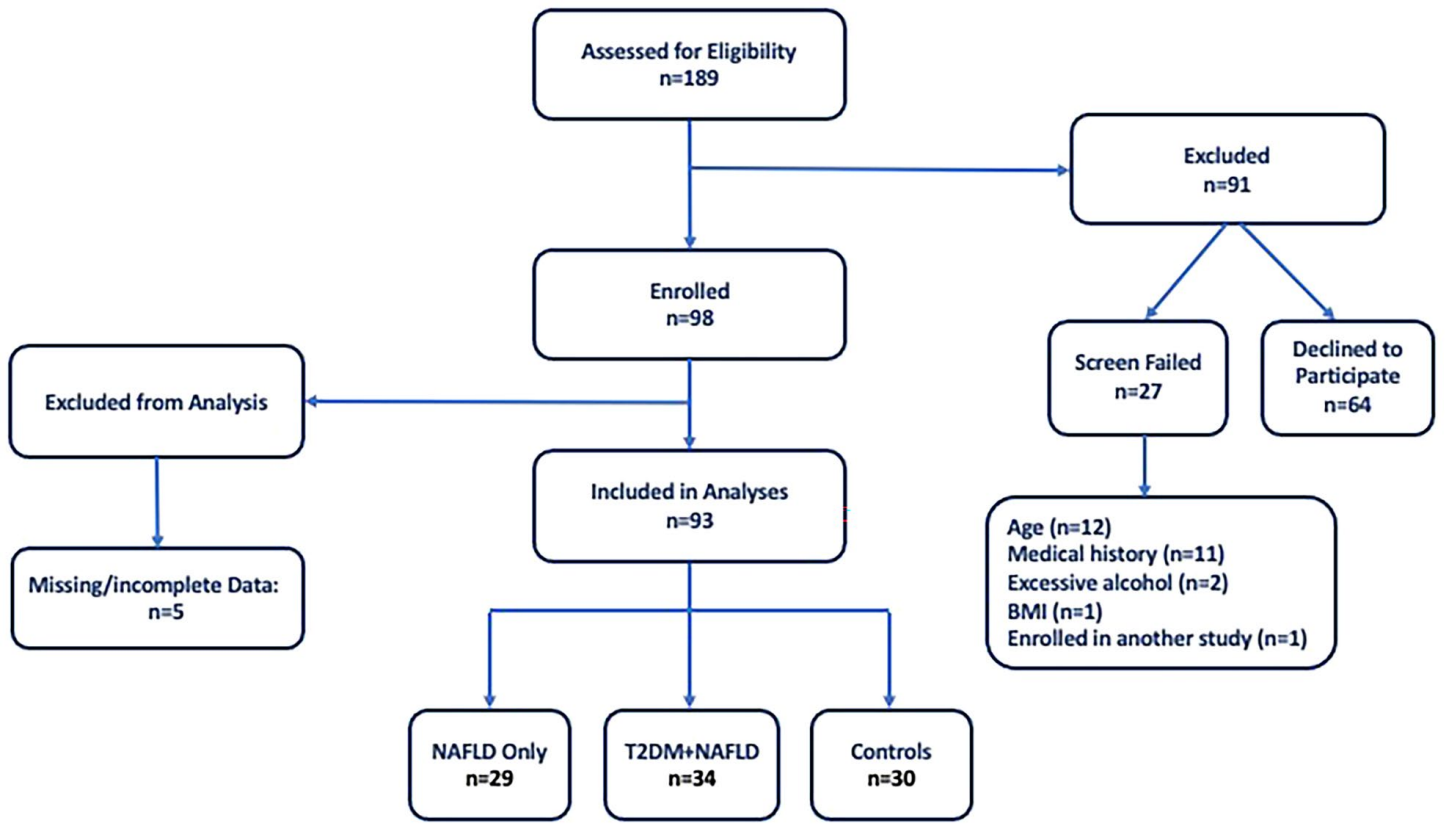
Participant flow diagram.

Individuals aged 25–69 were enrolled in a cross-sectional research study conducted at the Center for Liver Diseases at Inova Fairfax Hospital in Northern Virginia. Individuals with and without NAFLD and T2DM were recruited throughout the greater Washington D.C., Maryland and Virginia region from September of 2017 through March of 2020. Patients of the Center for Liver Diseases, identified from existing databases, were also recruited. Participants signed an informed consent document and the research was reviewed and approved by Inova Fairfax Hospital’s Institutional Review Board. A total of 93 participants were included for analysis and were divided into the following enrolment groups: (1) NAFLD only (*n* = 29); (2) T2DM + NAFLD (*n* = 34); and (3) controls (*n* = 30) (Figure 2).

Presence of NAFLD was determined by a radiologist via ultrasound classification and exclusion of other liver diseases (hepatitis B, C, autoimmune liver disease, copper and iron overload, and use of steatogenic medication as determined by review of available clinical records) and excessive alcohol consumption (greater than 14 units/week for males and 7 units/week for females) (one unit of alcohol is ½ pint of beer (285 mL), one glass of spirits (25 mL) or one glass of wine (125 mL)). Presence of T2DM was defined as having a prior diagnosis of T2DM or having a glycated haemoglobin A1C value ≥6.5 upon enrolment. Exclusion criteria were: history of traumatic brain injury, pregnant women or women who were less than 3 months post-partum, and any condition, which in the opinion of the investigator, would make the participants unsuitable for enrolment, or which could interfere with the participant completing the protocol.

Participants attended a one-time research visit at the Center for Liver Diseases clinic, where clinical, demographic, questionnaire and neurocognitive data were collected. To measure fine motor performance, the Grooved Pegboard Test (GPEG) was conducted. For GPEG testing, we used a standard GPEG device (Lafayette Instrument Company, Lafayette, IN) and followed published testing procedures [31]. The GPEG test consists of a manual pegboard in which examinees are instructed to: (1) insert pegs one at a time; (2) manipulate the peg, with one hand, in order to match the groove of the peg with the groove of the hole in the board; and (3) fill the rows in a given direction as quickly as possible without skipping any slots. This test is used to measure eye–hand coordination and motor speed, and there are two different trials: dominant hand and non-dominant hand. The score for GPEG is the time (in seconds) it takes for the examinee to finish placing all of the pegs into the board.

To further measure fine motor performance, the Trail Making Test (TMT) from the Delis Kaplan Executive Function System (DKEFS) battery was administered [32]. The Motor Speed condition of the TMT was used, as it is the purest measure of fine motor performance. In this condition, the examinee must connect circles together by drawing a line over an existing dotted line as quickly as possible.

The Human Activity Profile (HAP) Questionnaire [33] was administered in order to measure level of physical activity and it has previously been used in similar populations [34–36]. It consists of 94 items representing different activities, ranging from very easy (e.g. walking 30 yards) to very strenuous activities (e.g. running 3 miles). Participants were asked to indicate whether they still perform the activity, stopped doing the activity or never performed the activity. Three scores were calculated from the HAP: Maximal Activity Score (MAS), Average Activity Score (AAS) and Metabolic Equivalents Score (METS). MAS is the highest oxygen-demanding activity that the participant still performs representing the current maximum activity level of the respondent; AAS is the number of activities below the MAS that the participant reports as ‘stopped doing’ subtracted from the MAS representing the respondent’s average daily activity level; METS is the level of energy expenditure required to successfully engage in the highest oxygen-demanding activity. The HAP is free to use for research purposes with permission of the senior author of the instrument (D. Daughton).

Data were reported as mean ± standard deviation or number and percentages, as appropriate. A *p* value <.05 was considered statistically significant. To analyse statistically significant differences between the groups, one-way analysis of variance (ANOVA) was used for continuous data, chi-square tests were used for categorical data, and Kruskal–Wallis tests were used for ordinal data. Post hoc tests were used to determine differences between specific treatment groups when the omnibus ANOVA or Kruskal–Wallis test was statistically significant. Pearson’s correlation (for continuous data) and Spearman’s correlation (for ordinal data) analyses were used to investigate relationships between fine motor performance and physical activity. Partial correlation was then used to control for the impact of body mass index (BMI) on bivariate correlations. Statistical analyses were performed using SPSS, Version 27 software (IBM Corp., Armonk, NY).

To determine sample size, a power analysis was conducted. The main research question was to examine the relationship between fine motor performance and physical activity; therefore, the study was powered for a correlation analysis. To detect a moderate correlation (*r* = 0.05) [37] with an alpha (two-tailed) level of 0.05 and beta level of 0.20, 29 participants were required for each group. Once each group had at least 29 participants, recruitment was stopped [38].

## Results

### Demographic and participants characteristics

The participant characteristics are presented in Table 1. The study included 93 participants with 38 females (40.9%) and 55 males (59.1%). The average age of the participants was 52.2±12.1 years.

**Table 1.** Participant characteristics.

	All (*n*= 93)	T2DM + NAFLD (*n*= 34)	NAFLD (*n*= 29)	Controls (*n*= 30)	*p* Value, omnibus test
Age (years)	52.2 (12.1)	55.9 (9.7)	50.8 (11.9)	49.4 (14.0)	.075
Gender (female)	38 (40.9%)	15 (44.1%)	9 (31.0%)	14 (46.7%)	.422
BMI (kg/m^2^)	30.7 (6.6)	34.4 (5.3)^a^	32.8 (6.4)^b^	24.7 (3.0)^ab^	<.001
Race					
White (non-Hispanic)	58 (62.4%)	22 (64.7%)	20 (69.0%)	16 (53.3%)	.201
Hispanic	6 (6.5%)	3 (8.8%)	3 (10.3%)	0	
African American	11 (11.8%)	3 (8.8%)	1 (3.4%)	7 (23.3%)	
Asian	14 (15.1%)	6 (17.6)	4 (13.8%)	4 (13.3%)	
Other	3 (3.2%)	0	1 (3.4%)	2 (6.7%)	
Highest level of education					
High school diploma	13 (14.0%)	8 (23.5%)	3 (10.3%)	2 (6.7%)	.124
College degree	35 (37.6%)	13 (38.2%)	14 (48.3%)	8 (26.7%)	
Post graduate degree	44 (47.3%)	13 (38.2%)	12 (41.4%)	19 (63.3%)	
Alcohol consumption					
None	40 (43.0%)	16 (47.1%)	15 (51.7%)	9 (30.0%)	.264
1 drink per month	21 (22.6%)	7 (20.6%)	7 (24.1%)	7 (23.3%)	
1–2 drinks per week	15 (16.1%)	5 (14.7%)	1 (3.4%)	9 (30.0%)	
3–6 drinks per week	10 (10.8%)	2 (5.9%)	5 (17.2%)	3 (10.0%)	
1–2 drinks a day	4 (4.3%)	2 (5.9%)	1 (3.4%)	1 (3.3%)	
HbA1c (mmol/L)	6.0 (1.4)	7.2 (1.7)^ab^	5.4 (0.3)^a^	5.3 (0.3)^b^	<.001
Hypertension	44 (47.3%)	26 (76.5%)^ab^	12 (41.4%)^a^	6 (20.0%)^b^	<.001
Arthritis	20 (21.5%)	9 (26.5%)	6 (20.7%)	5 (16.7%)	.588
Depression (diagnosed)	12 (12.9%)	3 (8.8%)	3 (10.3%)	6 (20.0%)	.393
Hyperlipidaemia	44 (47.3%)	25 (73.5%)^ab^	12 (41.4%)^a^	7 (23.3%)^b^	.001

Matching superscript letters, within the same row, indicate statistically significant difference between groups. Values are expressed as either mean (standard deviation) or number (percentage). DKEFS Trail Making Test analysed using the Kruskal–Wallis test. Grooved Pegboard analysed using ANOVA.

Statistically significant clinical differences were found between the T2DM + NAFLD group and the control group for BMI, HbA1c, hypertension and hyperlipidaemia (*p*<.05, Table 1). In addition, statistically significant differences were found between the T2DM + NAFLD and NAFLD only group for HbA1c, hypertension and hyperlipidaemia. The only statistically significant difference between the NAFLD group and the control group was BMI (Table 1).

### Fine motor performance

Statistically significant differences between the T2DM + NAFLD and NAFLD only group were observed for GPEG dominant time and GPEG non-dominant time (*p*<.05, Table 2). Significant differences were also observed between the T2DM + NAFLD group and control group for the GPEG dominant time. For all of these, the T2DM + NAFLD group performed slower than their counterparts. However, there were no statistically significant differences between the groups for the TMT Motor Speed Scores.

**Table 2.** Motor and physical activity performance by T2DM and NAFLD status.

	All (*n*= 93)	T2DM + NAFLD (*n*= 34)	NAFLD (*n*= 29)	Controls (*n*= 30)	*p* Value, omnibus
Motor Speed Score for DKEFS Trail Making Test	11.3 (1.6)	10.7 (1.9)	11.7 (1.2)	11.5 (1.6)	.053
Grooved Pegboard Test dominant time	79.9 (14.8)	86.9^ab^ (15.1)	76.8^a^ (13.2)	75.1^b^ (13.3)	.002
Grooved Pegboard Test non-dominant time	86.1 (16.9)	92.0^a^ (19.9)	81.9^a^ (13.2)	83.7 (15.1)	.042
HAP Maximal Activity Score	83.7 (10.6)	79.6^a^ (11.7)	84.0 (9.1)	88.2^a^ (8.9)	.005
HAP Adjusted Activity Score	80.0 (12.8)	74.4^a^ (14.3)	81.0 (10.5)	85.3^a^ (10.9)	.003
HAP Metabolic Equivalents	7.9 (1.5)	7.2^a^ (1.5)	8.1 (1.3)	8.5^a^ (1.3)	.002

Values are expressed as means (standard deviation). Matching superscript letters, within the same row, indicate statistically significant difference between groups. DKEFS Trail Making Test analysed using the Kruskal–Wallis test. Grooved Pegboard and Human Activity Profile (HAP) analysed using ANOVA.

### Physical activity

Statistically significant differences between the T2DM + NAFLD group and control group were observed for HAP MAS, HAP AAS and HAP METS (*p*’s<.05, Table 2). The HAP scores for the control group were significantly higher (indicative of higher activity levels) than the scores for the T2DM + NAFLD group. There were no statistically significant differences in physical activity levels comparing the T2DM + NAFLD group to the NAFLD only group and comparing the NAFLD only group to the control group (Table 2).

### Relationship between fine motor performance and physical activity

**Figure 3.**
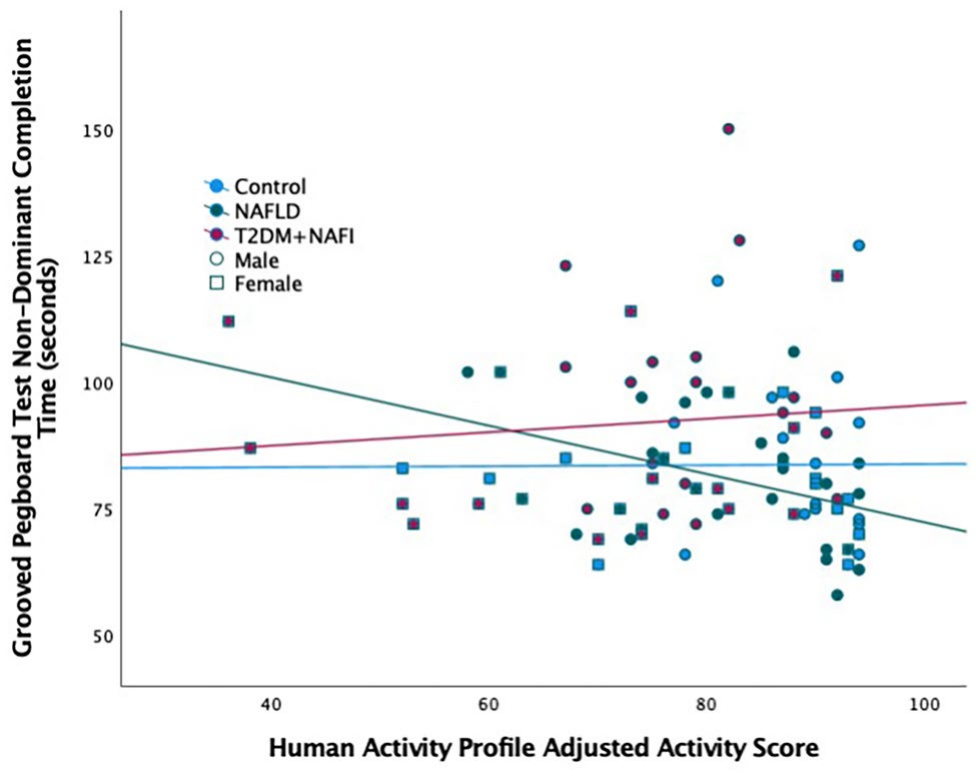
Relationship between physical activity and fine motor performance. Scatterplot of the Human Activity Profile Adjusted Activity Score and the Grooved Pegboard Test using the non-dominant hand. The three groups are represented by different colour markers and gender is represented by shape of the marker. The fit lines represent each of the group’s correlation (control: *r*=–0.077; NAFLD: *r*=–0.380; T2DM + NAFLD: *r* = 0.094).

The correlations between the TMT Motor Speed Score and HAP MAS and HAP AAS were statistically significant for the NAFLD only group (*p*’s<.05, Table 3). HAP AAS had a slightly higher correlation with the Motor Speed Score compared to HAP MAS for this group. The correlation between the GPEG non-dominant time and HAP AAS (Figure 3) and METS for the NAFLD group was also statistically significant (*p*’s<.05). HAP METS had a slightly higher correlation with GPEG non-dominant time compared to HAP AAS for the NAFLD group. These statistically significant correlations demonstrated that the higher levels of physical activity were related to better fine motor performance. No statistically significant relationships were found between fine motor performance and physical activity in the control and T2DM + NAFLD groups (*p* values>.05, Table 3). The correlations between fine motor performance and physical activity were then investigated further by controlling for BMI. The only correlation that remained statistically significant was the relationship between AAS and the Motor Speed Score for the NAFLD Group (*r* = 0.385, Table 4).

**Table 3.** Correlation between physical activity and fine motor performance.

		HAP Maximal Activity Score (MAS)	HAP Adjusted Activity Score (AAS)	HAP Metabolic Equivalents (METS)
Control	Motor Speed Score for DKEFS Trail Making Test	–0.108	–0.077	0.015
	Grooved Pegboard Test Dominant Time	–0.019	0.052	–0.232
	Grooved Pegboard Test Non-Dominant Time	–0.061	0.007	–0.265
NAFLD	Motor Speed Score for DKEFS Trail Making Test	0.377*	0.436*	0.356
	Grooved Pegboard Test Dominant Time	–0.324	–0.248	–0.325
	Grooved Pegboard Test Non-Dominant Time	–0.374	–0.380*	–0.433*
T2DM + NAFLD	Motor Speed Score for DKEFS Trail Making Test	0.140	0.180	0.166
	Grooved Pegboard Test Dominant Time	0.246	0.120	0.106
	Grooved Pegboard Test Non-Dominant Time	0.183	0.094	0.081

DKEFS Trail Making Test compared using Spearman’s correlation coefficient. Grooved Pegboard compared using Pearson’s correlation coefficient.

* Correlation is statistically significant at .05 level (two-tailed).

**Table 4.** Partial correlation between physical activity and fine motor performance controlling for body mass index.

		HAP Maximal Activity Score (MAS)	HAP Adjusted Activity Score (AAS)	HAP Metabolic Equivalents (METS)
Control	Motor Speed Score for DKEFS Trail Making Test	0.076	0.146	0.089
	Grooved Pegboard Test Dominant Time	–0.058	0.025	–0.268
	Grooved Pegboard Test Non-Dominant Time	–0.090	–0.012	–0.291
NAFLD	Motor Speed Score for DKEFS Trail Making Test	0.242	0.385*	0.253
	Grooved Pegboard Test Dominant Time	–0.105	0.023	–0.124
	Grooved Pegboard Test Non-Dominant Time	–0.081	–0.062	–0.192
T2DM + NAFLD	Motor Speed Score for DKEFS Trail Making Test	0.113	0.058	0.004
	Grooved Pegboard Test Dominant Time	0.300	0.166	0.147
	Grooved Pegboard Test Non-Dominant Time	0.298	0.204	0.181

* Correlation is statistically significant at .05 level (two-tailed).

## Discussion

This study aimed to determine the relationship between fine motor performance and physical activity for individuals at risk for developing MCI. In addition, we examined overall differences in fine motor performance and physical activity for a sample of 93 participants categorized by T2DM and NAFLD status. The T2DM + NAFLD group demonstrated slower performance on the GPEG compared to the control group. The T2DM + NAFLD group had consistently lower levels of physical activity than the NAFLD and control groups. Collectively, the data trend reflected that the NAFLD only group performed in between the T2DM + NAFLD and the control group for both physical activity and fine motor performance.

The relationship between physical activity and fine motor performance was statistically significant only in the NAFLD group. Specifically, the Motor Speed Score and the GPEG non-dominant time were statistically significantly correlated with physical activity, with the Motor Speed Score correlation with physical activity remaining statistically significant even after controlling for BMI.

Previous studies have also established the association between fine motor performance and physical activity [18–21]. Research findings revealed that an increased level of total daily activity and motor abilities can independently increase cognition and reduce dementia [19]. Moreover, physical activity can help alleviate the effects of white matter hyperintensity and motor function [20]. Physical activity can help individuals lead a healthy lifestyle [18–21] by protecting the brain from adverse neurobiological effects [20]. It can also improve motor abilities which provides a cognitive reserve to help maintain cognitive function [19]. In a study completed by Bossers et al. [18], a combination of aerobic and strength exercise was effective in decreasing the motor decline seen in dementia patients. Further research is still needed in order to understand the mechanisms that connect fine motor function and physical activity.

In the present investigation, only those with NAFLD demonstrated a statistically significant correlation between physical activity and fine motor performance. Previous work in populations with minimal hepatic encephalopathy (related to liver cirrhosis) have demonstrated deficits in fine motor performance [39]. The current population does not have liver cirrhosis, but does demonstrate the beginnings of this motor impairment. Important to identify this early to prevent the continued development of fine motor performance deficits. It is interesting to note, that the correlation between physical activity and fine motor activity in the individuals with T2DM + NAFLD was not statistically significant. A meta-analysis [40] found that dexterity, grip strength and pinch strength did not statistically significantly differ between those with diabetes and those without diabetes. In addition, individuals with diabetes are at increased risk of decreased physical activity that may reflect a link between the metabolic and mechanical functions of muscle [41]. This is in concert with the current findings, that individuals with NAFLD demonstrated a relationship between physical activity, but those with T2DM + NAFLD did not. Those with T2DM + NAFLD may have limited physical activity levels that are independent of fine motor performance. The physical activity level of individuals with only NAFLD may not have been impacted by the link between metabolic and mechanical functions of muscle specific to T2DM, keeping the relationship between physical activity levels and fine motor performance intact. Further research needs to investigate the impact of T2DM metabolic parameters on both physical activity and fine motor performance.

NAFLD is a growing public health problem and is the most common chronic liver disease in the world [10,11]. T2DM is frequently associated with NAFLD and research has shown that the prevalence of NAFLD in individuals with T2DM can range from 55% to 87% as there are many common risk factors shared between the two conditions, including hypertension, hyperlipidaemia and obesity [42,43]. An individual’s risk of developing diabetes is increased fivefold if they have NAFLD [44,45]. Therefore, it is important to understand potential differences in those with NAFLD and those with both NAFLD and T2DM.

Physical activity may be an effective early intervention for those with NAFLD, as it may prevent progression to developing T2DM + NAFLD. The T2DM + NAFLD group in the current investigation were less physically active and had slower fine motor performance than those with NAFLD. Physical activity can be implemented in an individual’s daily routine to help protect motor functions and has also been shown to prevent complications of NAFLD [14].

Exercise can also be used as an intervention for fat mobilization from the liver [25,46,47]. The combination of exercise and dietary interventions was effective in reducing intrahepatic triglycerides; however, the implementation of exercise only was also beneficial in reducing hepatic lipid levels [25]. Additional evidence also found a reduction in intrahepatic triglyceride to be proportional to the amount of weight loss that occurred [46]. Specifically, the amount of reduction is twofold greater when weight loss is achieved [46]. Therefore, engagement in physical activity can be promoted to mobilize hepatic fat, prevent the development of T2DM, and potentially protect fine motor performance.

There were limitations to the current study. The sample recruited was a community-based convenience sample; however, it was not representative of the general population, as it mainly included White (non-Hispanic) and highly educated individuals. Another limitation was the identification of NAFLD, which was determined by a radiologist using ultrasound. Ultrasound is a non-invasive, accessible and accurate tool in detecting NAFLD [48]; however, liver biopsy is the gold standard in diagnosing NAFLD and the most accurate in detecting fibrosis level. The procedure can be invasive and risky due to potential complications [49]. Lastly, while the findings of lower activity levels and slower fine motor performance are statistically correlated, we cannot comment on a causal relationship between the two findings. Nonetheless, recommendations for reducing the likelihood of developing T2DM is likely to be effective in also preserving fine motor performance.

## Conclusions

In summary, this study suggests that those with T2DM + NAFLD have lower levels of physical activity and slower fine motor performance compared to controls and those with NAFLD only. Reduced fine motor performance may be an early warning signal for developing MCI [16]. Further research needs to explore the mechanisms that impact fine motor performance and physical activity in those with NAFLD with or without T2DM in order to maintain functional levels and prevent comorbidities and early mortality. Individuals with NAFLD should be identified, advised and encouraged to engage in physical activity.

## Data Availability

The data from this study are available from author AAW (aweinst2@gmu.edu), upon reasonable request.
